# Improving Cancer Immunotherapy: Exploring and Targeting Metabolism in Hypoxia Microenvironment

**DOI:** 10.3389/fimmu.2022.845923

**Published:** 2022-02-24

**Authors:** Jinfen Wei, Meiling Hu, Hongli Du

**Affiliations:** School of Biology and Biological Engineering, South China University of Technology, Guangzhou, China

**Keywords:** hypoxia, metabolic reprogramming, cancer immunotherapy, single-cell analysis, cell subtypes

## Abstract

Although immunotherapy has achieved good results in various cancer types, a large proportion of patients are limited from the benefits. Hypoxia and metabolic reprogramming are the common and critical factors that impact immunotherapy response. Here, we present current research on the metabolism reprogramming induced by hypoxia on antitumor immunity and discuss the recent progression among preclinical and clinical trials exploring the therapeutic effects combining targeting hypoxia and metabolism with immunotherapy. By evaluating the little clinical translation of the combined therapy, we provide insight into “understanding and regulating cellular metabolic plasticity under the current tumor microenvironment (TME),” which is essential to explore the strategy for boosting immune responses by targeting the metabolism of tumor cells leading to harsh TMEs. Therefore, we highlight the potential value of advanced single-cell technology in revealing the metabolic heterogeneity and corresponding phenotype of each cell subtype in the current hypoxic lesion from the clinical patients, which can uncover potential metabolic targets and therapeutic windows to enhance immunotherapy.

## Introduction

Since the Food and Drug Administration (FDA)-approved immune checkpoint blockade (ICB) in 2011, immunotherapy has achieved unprecedented advances in clinical treatment among various cancer types (1). However, a large proportion of patients still do not get a clinical benefit (1, 2). There are mainly two categories underlying resistance to immunotherapy, including host heterogeneous factors (such as age, gender, personal diet, drug use, lifestyle) (3, 4) and the host internal factors containing tumor cell genome and composition characteristics of the tumor microenvironment (TME) (such as the cytokine, metabolic, and cellular interaction) (5). The TME is an extensively discussed component, as TME is composed of cancer cells, stromal cells, and extracellular matrix (ECM), as well as soluble molecules in ECM (6). Antitumor function of immune cells is influenced by characteristics of the TME, such as crosstalk with stromal cells (7), concentrations of inflammatory factors and chemokines (8), degree of hypoxia (9), accumulation of harmful metabolites, and nutrient levels (10). Therefore, to improve the antitumor immunity, it is necessary to understand the complex biological characteristics of TME and the corresponding cell state of each subtype.

Among the characteristics of TME, hypoxia is a prevalent resistance factor to immunotherapy, which contributes immune escape (11) and is often accompanied by metabolism reprogramming and acidic metabolite efflux of cells within hypoxia TME (12). Thus, in addition to insufficient oxygen, the effects induced by hypoxia also cooperatively contribute to dysfunction of antitumor immunity (13). Therein, metabolism activity not only provides energy for survival of immune cells but also is critical for antitumor functions of immune cells through a variety of mechanisms (14). Over the past years, studies on metabolism of immune cells have been revealed and well-reviewed (15). For example, many studies highlighted the upregulation of glycolysis (16) as well as mitochondrial metabolism (17) as the hallmark of T-cell activation. Besides, proliferating immune cells, including activated T cells, rely on glutamine (18), serine (19), tryptophan (20), cysteine (21), and other amino acid metabolism to support protein and nucleotide synthesis. However, the increased uptake and activated metabolism of glucose and glutamine in tumor cells cause the scarcity of these nutrients in the TME, resulting in the loss of metabolic activity of effector T cells and promoting exhaustion phenotype (15). Immunosuppressive cells including regulatory T cells (Tregs), M2-like tumor-associated macrophages (TAMs), and myeloid-derived suppressor cells (MDSCs) could use fatty acid oxidation (FAO) to provide cell energy and further maintain immune suppression on effector T cells under hypoxia and nutrition-deprived condition (22). Ultimately, these metabolic changes in immune effector cells and immunosuppressive cells can impede the efficacy of antitumor immune responses. It seems like regulating hypoxia-induced metabolism of tumor cells and immunosuppressive cells could improve the antitumor immune response and inhibit tumor growth. Therefore, it is urgent that exploring cellular metabolic plasticity in each cell type under current TME, regulating critical metabolic pathways that exacerbate TME or damage the immunity of effector cells, is the primary task of improving immune response and immunotherapy (23).

Recently, technological advances such as single-cell RNA sequencing (scRNA-seq), flow cytometry-based methods, can clearly distinguish individual cells in the TME (24), which could reveal the metabolic heterogeneity of various types of cells in the current TME and further help discover potential metabolic checkpoints for tumor immunotherapy (25). In this light, this review focuses on discussing the critical impacts of metabolism reprogramming and acidosis TME on immune function under hypoxia conditions, discussing the emerging strategy of targeting the critical metabolic pathway and hypoxia to enhance immunotherapy, highlighting novel discoveries delineating the heterogeneity of cells within TME based on single-cell approaches and prospecting the view that reveals the immunometabolism heterogeneity from the perspective of clinical patients by “understanding and regulating cellular metabolic plasticity under the TME” *via* advanced single-cell technology.

## Antitumor Immunity Dysfunction Caused by Metabolic Reprogramming in Hypoxia Tumor Microenvironment

**Figure 1:**
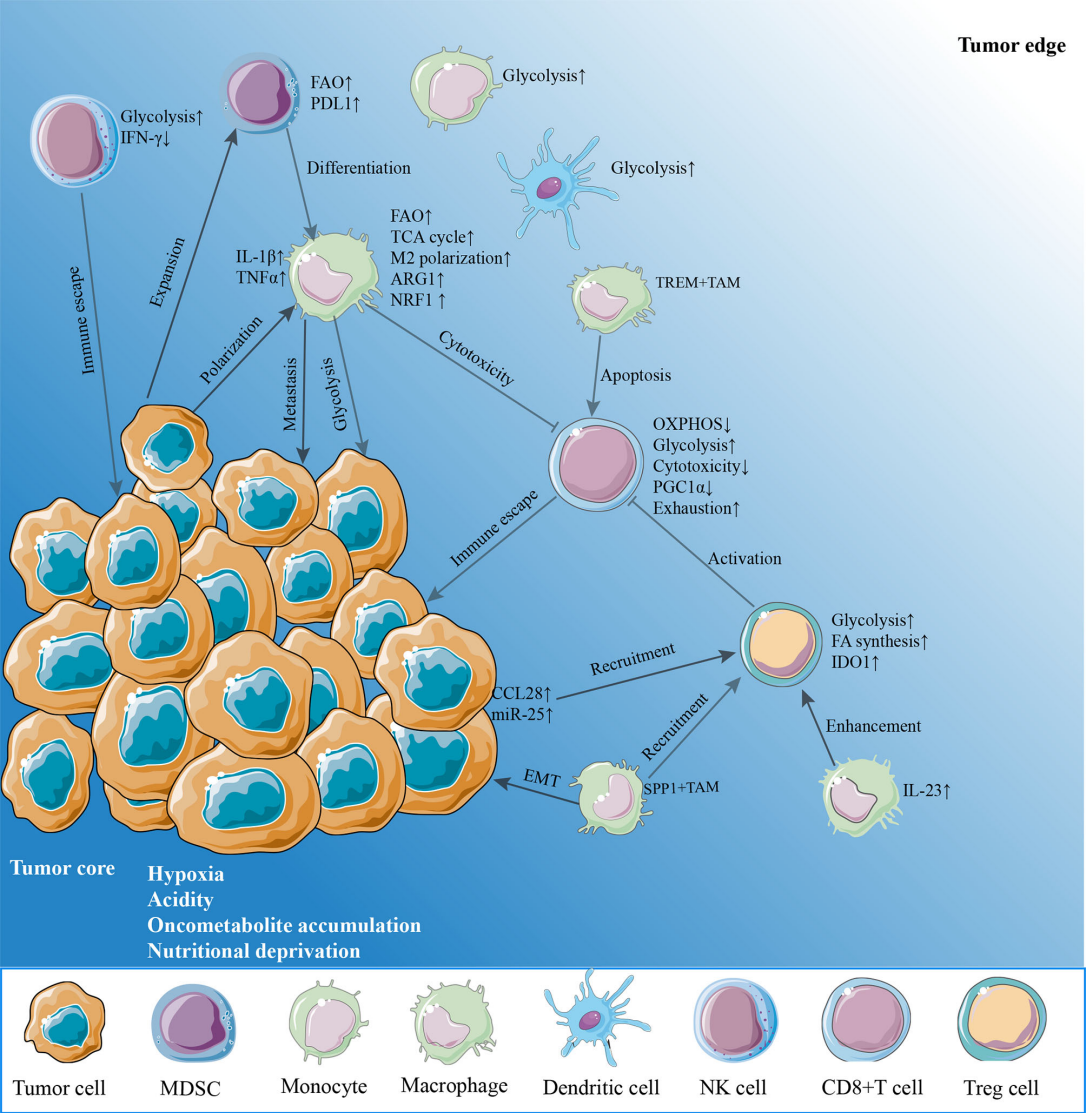
The hypoxia microenvironment. The hypoxia tumor microenvironment (TME) suppresses antitumor immunity on effector CD8^+^ T cells and natural killer (NK) cells through enhancing metabolic stress in a variety of mechanisms. Hypoxia, acidity, and nutrient deprivation are the main characteristics of the TME. Cancer cells upregulate glycolysis, oxidative phosphorylation (OXPHOS) to support rapid proliferation, resulting in an oxygen-reduced, glucose-deprived, and lactate-enriched microenvironment. This glucose-deprived TME restricts glycolysis and OXPHOS in tumor-infiltrating lymphocytes such as CD8^+^ T cells and NK cells. Hypoxia also damages the mitochondrial function by reducing PGC1α expression of CD8^+^ T cells, leading to the exhausted phenotypes and reduce the release of cytotoxic factors including IFN-γ. By contrast, regulatory T cells (Treg) increase fatty acid synthesis and glycolysis, while myeloid-derived suppressor cells (MDSCs) and tumor-associated macrophages (TAMs) enhance fatty acid oxidation to provide energy. Obviously, these immunosuppression cells would survive by adjusting their metabolisms and further enhance immunosuppressive phenotype. Reciprocally, tumor cells promote macrophage polarization to an M2-like phenotype. TREM+TAMs expand in hypoxia TME, leading to CD8^+^ T cell apoptosis. SPP+TAMs expand in hypoxia TME and promote epithelial–mesenchymal transition (EMT) of cancer cells and also recruit Tregs to TME. Furthermore, other myeloid cells, monocytes and dendritic cells, prefer glycolysis in the TME. IFN-γ, interferon-gamma; PD-L1, programmed cell death receptor ligand 1; IL-1β, interleukin-1beta; TNFα, tumor necrosis factor alpha; ARG1, arginase 1; NRF1, nuclear factor erythroid-derived 2-related factor 1; TREM, triggering receptor expressed on myeloid cell; PGC1α, peroxisome proliferator-activated receptor gamma coactivator 1 alpha; CCL28, chemokine CC-chemokine ligand 28; IDO1, indoleamine 2,3-dioxygenase 1; IL-23, interleukin-23.

Although tumors are heterogeneous, most solid cancers exhibit hypoxia conditions compared to normal tissues (26). In addition to the effect on tumor cells, it has been revealed that hypoxia influences the immune function through disrupting or altering metabolism in immune cells infiltrated in TME due to hypoxia-inducible factor-1 (HIF-1)­dependent or -independent effects. In this section, we describe the metabolic landscape of the TME that play roles to destroy antitumor immunity and describe targeting effects of specific metabolism and hypoxia that restore the immunity (**Figure 1**).

### Metabolism Alteration Dampens the Function of Immune Effector Cells in Hypoxia Condition

Immune effector cell infiltration is a key indicator of effective anti-immune response. Preclinical research shows that the effector T-cell infiltration is negatively related to hypoxia level in prostate cancer, and they found that using hypoxia-activated prodrug TH-302 could guide invasion of T cell to TME (27). Besides, the accumulating evidence suggests that a high proportion of T cells are exhausted and localized in the TME across various cancer types, resulting in immune dysfunction in cancer patients (28, 29). As hypoxia is the main characteristic of TME, elucidating whether hypoxia regulates T-cell exhaustion and which signal response to hypoxia is vital to develop novel strategies to activate the immune response. Interestingly, two recent pieces of research indicate that mitochondrial dysfunction and dynamics induced by continuous hypoxia stress lead to the appearance of exhaustion phenotype and upregulation of exhaustion-related genes in CD8^+^ T cells *in vitro* (30, 31). Relieving hypoxia in TME by reducing oxygen consumption of engineered tumor cells or regulating hypoxia response factor by overexpression of peroxisome proliferator-activated receptor gamma coactivator 1 alpha (PGC-1α) in T cells results in a significantly reduced subset of T-cell exhaustion in the melanoma mouse model (31). Inhibiting the signal that result in hypoxia TME and mitochondrial dynamics in T cells recovers the mitochondrial metabolism of T cells, prevents T-cell exhaustion, and further decreases tumorigenesis in nasopharyngeal carcinoma mouse model (30). Similarly, a terminally exhausted phenotype of T cells is generated under hypoxia conditions *in vitro* (32). As cells that adapt to hypoxia tend to upregulate and stabilize HIF, it was found that expression of genes related to T-cell exhaustion is highly associated with HIF1A expression in glioma patients, indicating that HIF1A may also signal the responding hypoxia to regulate T-cell exhaustion status (33). Growing tumor increases the oxygen demand and causes a harsh hypoxia TME; however, oxygen is also crucial for the T cells to survive, implying that oxidative metabolism of tumor cells may be a potential target to improve antitumor immunity (34). Relieving symptoms of tumor hypoxia by metformin treatment brings an antitumor effect through inhibiting cyclic AMP pathway on γδ T cells (35). Peroxisome proliferator-activated receptor alpha (PPAR-α) agonist could enhance fatty acid catabolism of T cells under hypoxia and low-glucose TME, thus improving the immune response (36). Besides, hyperoxic breathing is another way used to enhance T-cell infiltration and improve lung tumor survival in mice (37).

Natural killer (NK) cell is reported to secrete fewer cytokines including interferon-gamma (IFN-γ) in the hypoxia TME (38). Consistently, compared with the control group, HIF1A-deficient NK cells display more oxidative phosphorylation (OXPHOS) but less glycolysis, along with significantly producing IFN-γ, when exposed to prolonged hypoxia (39), indicating that NK cells might use the full potential of OXPHOS for their antitumor function. Given this critical dependence on OXPHOS for the cytotoxic effect of T/NK cells, reducing the oxygen consumption of tumor cells or downregulating hypoxia-dependent downstream metabolic pathways of immune cells will increase oxygen utilization and OXPHOS in T/NK cells. Of note, the *in vivo* study about the effect of hypoxia on T/NK cell’s function is scarce, and further study is needed to assess whether metabolism dysfunction is the pivotal factor to dysfunction of these cytotoxic lymphocytes under the prevalence of hypoxic regions in tumors.

### Metabolism Alteration Drives Function of Immunosuppressive Cells in Hypoxia Condition

Immunosuppressive cells are revealed to develop self-interest strategies instigated by tumor cells to survive in hypoxia TME and further block the immune response, including metabolic adaptation and adjustment under hypoxia conditions.

Various studies indicate that Tregs, rather than CD4+ effector T cells, are recruited and activated in the hypoxia zone of colon cancer (40), melanoma (41), and lung cancer (42) to enhance immunosuppression in TME. Of note, HIF1α is also the key metabolic sensor, and this function is largely contributing to metabolism adaptability under hypoxia conditions. HIF1α activates glycolysis instead of OXPHOS in Tregs, leaving FAO to support Treg activity and suppressing effector T cell proliferation within the hypoxic zone (43). Inhibition of lipid uptake in Tregs could increase the antitumor function of CD8^+^ T cells and prolong the survival in the mouse model of brain tumors (43). Interestingly, Tregs utilize an HIF1α-driven metabolic switch only under hypoxia TME, which reflected that activating FAO and upregulating HIF1α are the metabolism adjustments of Tregs under hypoxia TME and may be targets to improve antitumor immunity. In conformity with the earlier study, HIF1α knockout prevents Tregs to enter the TME and further improves the survival of the brain mouse model (44). HIF1α activates glutamine-deprived macrophages to secrete interleukin (IL)-23, which enhances Treg immunosuppressive function in a clinical sample and mouse model of kidney cancer study (45).

As TAMs tend to accumulate in hypoxic regions, hypoxia promotes the polarization of TAMs to the immunosuppressive phenotypes (15). The hypoxia-activated Seven in absentia homolog 2 (SIAH2)-nuclear factor erythroid-derived 2-related factor 1 (NRF1) signal axis suppresses mitochondrial function and induces immune response of protumor in macrophages, and regulation of NRF1 in macrophages could inhibit polarization of TAMs and restrain tumor maintenance in breast cancer (46). Based on the mouse model, hypoxia could enhance OXPHOS and M2-like polarization phenotype in TAMs through tumor-secreted exosomes across melanoma, squamous skin carcinoma, and lung cancer (47). Triggering receptor expressed on myeloid cell (TREM)-1+ TAMs are abundant in hypoxia TME and undermine the effect function of CD8^+^ T cells and cause the apoptosis of T cells in liver cancer patients (48). Pharmacological inhibition TREM-1 revokes the immunosuppression by abrogating Treg recruitment and enhancing the T-cell cytotoxic function, further eliminating the programmed cell death receptor ligand 1 (PD-L1) blockade resistance (48). IL-1β, highly secreted by macrophages, enhances tumor cell metastasis through HIF1α signals under hypoxic TME in the liver (49) and breast mouse cancer model (50). Conversely, TAMs could exacerbate hypoxia levels and glycolysis of tumor cells (51). Undoubtedly, removing TAMs in the TME could lead to increases in infiltration and antitumor immunity of T cells in the mouse model (51). MDSCs turning differentiation to TAMs is regulated by HIF1α under hypoxia in several solid turning models (52). Hypoxia induces 5'-adenosine mono phosphate (5’-AMP) secreted by tumor cells in the extracellular space, thus promoting the maintenance of MDSCs and further enhancing immunosuppressive activities in liver cancer (53). PD-L1 is upregulated in MDSCs, and blocking PD-L1 decreases IL-6 and IL-10 expression in MDSCs and abrogates the suppression to CD8^+^ T cells under hypoxia conditions (54). In sum, hypoxia is the major factor to promote immunosuppressive cell function and polarize TAM to anti-inflammatory phenotype, thus regulating metabolism, and hypoxia may be a realizable approach to prompt antitumor immunity.

### Effect of Extracellular Acidification on Immune Dysfunction in Hypoxia Condition

The intrinsic effect of hypoxia cannot always be induced by insufficient oxygen directly. Understanding the integration of insufficient oxygen-mediated responses together with other hypoxia-driven effects is key to revealing the biology of immune cells in hypoxia. CD8^+^ T cells become an energy phenotype and lost effect function in a low pH medium *in vitro*, and regulating the pH by pharmaceutical treatment could increase the therapeutic potential of adoptive immunotherapy in melanoma-bearing mouse model (55). Immune effector cells lost antitumor function along with diminishing IFN-γ production in high lactic acid production mice, leading to tumor immune escape in melanoma (56). However, unlike effector T cells, Tregs can survive in acid and lactate-rich TME, further suppressing the effector T-cell function (57, 58). Studies have shown that lactate derived from tumor cells can induce the expression of vascular endothelial growth factor (VEGF) and arginase 1 (ARG1) through the HIF1A signaling pathway and promote the polarization of TAM to M2-like (59, 60). Similarly, lactate stimulates macrophage M2-like polarization (61), alters pro- to the anti-inflammatory response of macrophage *via* G-Protein coupled receptor 81 (GPR81)-mediated Yes1 associated transcriptional regulator (YAP) inactivation (62), and subsequently promotes T cell apoptosis in an *in vitro* study (63).

Based on the universality of hypoxic regions and acidification in tumors and the lasting impact of hypoxia on anti-immune response, further research is warranted to focus on the heterogeneous responses of different cells induced by hypoxia. That said, various cell-specific hypoxia-induced metabolic pathways and metabolic flexibility within a specific immune cell subpopulation and functional state need to be understood and presented in various cancers, which helps to explore ways for targeting metabolism to improve the immune response.

## Targeting Metabolism and Hypoxia to Enhance Immunotherapy Response

Overall, the effect of antitumor immunity is affected by hypoxic environment and metabolic disorders; therefore, regulating metabolism or hypoxia for cooperation immunotherapy effect deserves much more attention. Besides, various studies show that checkpoint signaling is regulated by metabolism and hypoxia and *vice versa* also affects (11, 64). Therefore, the prospect of combining metabolic inhibitors or targeting hypoxia treatment, with checkpoint inhibitors, is expected to enhance the efficacy of checkpoint blocking (**Table 1**).

**Table 1:** Outcomes of preclinical studies with combined immune checkpoint inhibitors and metabolic inhibitors across various cancer types.

Cancer types	Treatment	Animal Model	Outcome	References
Melanoma	Anti-PD-1+Inhibition lipid metabolism in cancer cells	C57BL/6J mice	Increase sensitivity to T cell-mediated killing	(65)
Melanoma	Anti-PD-1+GLUT1 knockdown in tumor cells	C57BL/6J mice	Increase the immune activity and overall survival	(34)
Melanoma	Anti-PD-1+Metformin	C57/BL6, OT-I mice	Improve intratumoral T-cell function and tumor clearance but lose sensitivity in aggressive tumors	(66)
Melanoma	Anti-PD-L1+IDO inhibitor	C57BL/6 mice	Enhance antitumor immune response, decrease tumor volume, increase mouse survival	(67)
Melanoma	Anti-PD-1+MCT inhibitor	C57BL/6J or C57BL/6N mice	Delay tumor growth	(68)
Melanoma	Anti-PD-1+Glutamine antagonist	C57BL/6 mice	Delay tumor growth, prolong animal survival time	(69)
Melanoma	Anti-PD-1+SREBP inhibitor	C57BL/6 mice	Reduce tumor growth and prolong survival in B16-bearing mice	(70)
Melanoma	Anti-CTLA-4, anti-PD-1+Bicarbonate supplementation	C57BL/6 mice	Decrease tumor growth	(71)
Melanoma	Anti-PD-1+Nanoparticle containing MCT1 inhibitor	C57BL/6 mice	Prolong long-term survival	(72)
Breast cancer	Anti-PD-1+LDH inhibitor	BALB/c mice	Inhibit tumor growth	(73)
Breast cancer	Anti-CTLA-4+LDHA-KD	BALB/cAnN mice	Prolong the survival outcomes	(74)
Breast cancer	Anti-PD-1+Glutamine antagonist	BALB/cJ mice	Enhance the efficacy of immune checkpoint blockade, reduce tumor growth	(75)
Breast cancer	Anti-PD-1/PD-L1+BO1-CSF2–KO tumors	C57BL/6J mice	Decrease the tumor growth and the rate of metastasis	(76)
Colon cancer	Anti-PD-1+Folate Pathway Inhibitor	BALB/c and C57BL/6 mice	Increase in tumor cell killing	(77)
Colon cancer	Anti-PD-1+PPARγ coactivator	C57BL/6N and BALB/c mice	Enhance antitumor immunity, improve the efficacy of PD-1 blockade	(78)
Colon cancer	Anti-PD-1+Treatment with pH-modulating injectable gel (pHe-MIG)	C57BL/6 mice	Lead to tumor clearance	(79)
PDAC	Anti-PD-1+GFAT1 inhibitor (DON)	C57BL/6 mice	Reduce tumor weight and tumor volume	(80)
Osteosarcoma	Anti-PD-L1+L-arginine supplementation	BALB/c mice	Prolong survival of osteosarcoma-bearing mice	(81)
Colon and lung cancer	Anti-PD-1+Mitochondrial activators	C57BL/6 or BALB/c mice	Suppress tumor growth	(82)
Melanoma and colon cancer	Adoptive T-cell transfer immunotherapy+Interleukin-10-Fc	C57BL/6 (C57BL/6J) mice	Revitalize terminally exhausted T cells, eradicate solid tumors	(83)
Melanoma and lung cancer	Anti-PD-L1+FATP2 inhibitor lipofermata	C57BL/6 mice	Enhance anti-PD-L1 tumor immunotherapy and delay tumor progression	(84)
Pan-cancer	Anti-PD-1+Nanoparticle containing PDK1 inhibitor	BALB/c mice	Enhance cytotoxic T-cell infiltration, decrease the tumor volume	(85)
Pan-cancer	Anti-PD-1+Nanoparticle targeting knockdown LDHA	BALB/c, C57BL/6 mice	Inhibit tumor growth	(86)
Pan-cancer	Anti-PD-L1+Inosine supplementation	C57BL/6 mice	Delay tumor growth, prolong animal survival time	(87)

PD-1, Programmed cell death 1 (PD-1); GLUT1, Glucose transporter type 1; PD-L1, Programmed cell death 1 ligand 1; IDO, Indoleamine 2,3-dioxygenase; MCT, Monocarboxylate transporter; SREBP, Sterol regulatory element binding transcription factor; CTLA-4, Cytotoxic T-lymphocyte associated protein 4; MCT1, Monocarboxylate transporter1; LDHA, Lactate dehydrogenase A; CSF2, Colony stimulating factor 2; PPARγ, Peroxisome proliferator-activated receptor gamma; GFAT1, Glutamine--fructose-6-phosphate transaminase 1; FATP2, Fatty acid transport protein 2; PDK1, Pyruvate dehydrogenase kinase 1.

### Targeting Cancer Cell Metabolism to Enhance Immunotherapy

Metabolism reprogramming in tumor cells has been suggested as the characteristic for evaluating immunotherapy under hypoxia. For example, Harel et al. (65) find that high mitochondrial metabolism in melanoma cells is associated with the better effect of ICB treatment as the higher antigen presentation in tumor cells. The inconsistent results have been revealed by other studies, as mitochondrial metabolism in tumor cells results in hypoxia, which damages the antitumor function of effector T cells. OXPHOS metabolism of cancer cells is associated with T-cell exhaustion and poor response to Programmed cell death 1 (PD-1) blockade immunotherapy in melanoma due to high oxygen consumption resulting in hypoxia in the TME (34). Inhibiting OXPHOS by Ndufs4 knockdown in tumor cells or metformin treatment has reduced hypoxic environments and enhanced PD-1 blockade efficacy (34, 66). Consistent with the above study (35), these studies show that metformin treatment could reduce oxygen consumption in cancer cells and relieve hypoxia in metformin-treated animal model, further enhancing antitumor immunity. Besides, metformin inhibits mitochondrial complex I of cancer cells in the micromolar range, which is a comparable plasma steady-state concentration of metformin to diabetics who received standard doses of metformin (88–90). However, metformin may inhibit tumor cell proliferation through immune-mediated mechanisms because metformin directly improves effector T-cell function *in vivo*. Combining metformin with an anti-PD-1 treatment directly activates CD8^+^ T cells and boosts IFN-γ secretion, leading to the decreased glycolysis and OXPHOS of tumor cells compared with anti-PD-1 treatment alone (91). It is found that metformin-treated animals reduce the PD-L1 stability on cancer cells, then increase the activity of cytotoxic T lymphocytes (92). Above all, metformin may affect the antitumor mechanism of immune cells through multiple mechanisms, and more research on pharmacokinetics and mechanisms is further needed to better define the effects of metformin on the cancer immune system. Regarding details of applying metformin in cancer therapy in more studies, we refer and recommend the more excellent and comprehensive reviews on this topic (93, 94).

As glycolysis is highly needed for tumor cells, inhibiting glycolysis in tumor cells could augment the anti-PD-1 response without affecting T-cell function in melanoma (68) and breast cancer mouse model (73). Carbonic anhydrase IX (CAIX), upregulated by HIF to activate glycolysis, contributes to cancer cell growth by enhancing the efflux of lactate under hypoxic conditions (95). Pharmacological inhibition of CAIX combined with immunotherapy may be a potential strategy for the treatment of hypoxia tumors (96).

Effector T cells also require another metabolism, like methionine, glutamine, and folate. High uptake of methionine by tumor cells leads to methionine deficiency in TME, and treatment combination of an inhibitor of methionine transporters and anti-PD-L1 significantly inhibits tumor growth compared with anti-PD-L1 treatment alone (97). Targeting glutamine-utilizing enzyme enhances the anti-PD-1 therapy effect by increasing CD8^+^ T cell infiltration (80). Pharmacologically inhibiting glutamine metabolism by antagonist JHU083 enhances the effect of anti-PD-1 treatment in immunotherapy-resistant tumors by regulating the metabolism of tumor cells on the Trichloroacetic acid (TCA) cycle and amino acids (75). Inhibiting folate metabolism synergizes anti-PD-1 treatment by hampering tumor cell survival and increasing the mitochondrial metabolism of T effector cells (77).

### Targeting Immune Cell Metabolism to Enhance Immunotherapy

Conversely, activation of immune cellular metabolism is a straightforward strategy to enhance immunotherapy. For example, pharmacological activation of Mechanistic target of rapamycin (mTOR) and their downstream factors wound synergize anti-PD-1 treatment in the colon cancer mouse model (82). Activation of OXPHOS by IL-10 protein can restore the vitality of terminally exhausted T cells, potentiate the anticancer function, and prolong the survival time when combined with anti-PD-1 treatment (83). As we know, T cells will preferentially select glycolysis for energy and immune function (98, 99); however, due to glucose deprivation by tumor cells, T cells with limited access to glucose switch to OXPHOS (23). Hypoxia is common in solid cancer that suggests that there needs to be another strategy to support T-cell metabolism in the nutrient- and O_2_-limited TME. The fatty acid is another source for supporting mitochondrial respiratory capacity to enhance T-cell infiltration level as well as the antitumor function in the hypoxia and insufficient glucose supply TME. PPAR agonist is observed to induce FAO in T cells and enhance PD-1 blockade therapy in melanoma (36), which suggest that increasing catabolism of fatty acids in CD8^+^ T cells may enhance antitumor ability in the glucose deprivation and hypoxia TME. Supplementation of nutrients in the TME is also a way to activate an immune response to enhance immunotherapy. Inosine (87) and L-arginine (81) supplementation combined with anti-PD-L1 treatment could increase the number of CD8^+^ T cells expressing cytotoxic factors and improve survival compared with anti-PD-L1 alone in the mouse models. Ideally, exploring and targeting specific metabolism enable to suppress tumor growth and increase immune cell activity, which will improve immunotherapy. It has recently been shown that combining glutamine antagonist and anti-PD-1 immunotherapy dramatically improves antitumor effects compared with anti-PD-1 therapy alone (69), revealing the bidirectional effect of glutamine deprivation with increasing OXPHOS and cell activity of CD8^+^ T cells and decreasing the glycolysis in cancer cells.

Tregs would lose function in the absence of glucose, and combining glycolysis inhibition with CTLA-4 blockade enhances the overall survival in the mouse model (74). Inhibition of fatty acid metabolism in MDSCs could enhance anti-PD-L1 tumor immunotherapy (84) and adoptive T-cell therapy (100), inducing a significant antitumor effect. Blocking fatty acid synthesis in M2-like TAMs would lead to mitochondrial damage to the M2 macrophage phenotype, relieve the immunosuppressive environment, and further enhance anti-PD-1 immunotherapy (70). Inhibiting arginase 1 (ARG1) expression in TAMs would enhance immune therapy including anti-PD-1 and anti-CTLA4 in breast cancer (76). Moreover, short-chain fatty acids in TME limit the capacity of DC to stimulate T cells and restrict the antitumor effect in CTLA-4 blockade treatment (101).

The above partial studies only emphasized the regulation of metabolism on immune response without assessing hypoxia. The future study needs to characterize and evaluate the metabolic inhibitory effect to trigger immunotherapy response in hypoxic tumors.

### Reducing Hypoxia and Acidity to Enhance Immunotherapy

In addition to targeting metabolic pathways activated by hypoxia, directly targeting hypoxia and acidity is also an effective way to increase sensitivity to immunotherapy response. Relieving hypoxia using hypoxia-activated prodrug TH-302 combined with ICB treatment could reduce MDSC density, thus increasing T-cell infiltration and restoring the antitumor effect (27). As mentioned above, acidic TME impairs the function of T cells; adding bicarbonate monotherapy to neutralize would increase T-cell infiltration and enhance antitumor responses in tumor models (71). NaHCO_3_-loaded Pluronic F-127 effectively alleviates extracellular tumor acidity and increases the anti-PD-1 treatment (79).

### Application of Nanomaterials Loading Metabolism Drugs Under Hypoxic and Acid Tumor Microenvironment to Enhance Immunotherapy

Due to extreme hypoxia and acidic conditions within the TME, using new nanomaterials can accurately solve the problem of drug delivery in the hypoxic and acidic niche. Combined anti-PD-1 inhibitor with nanoparticle containing a glycolysis inhibitor can significantly enhance several T cells and enhance therapy effect (85). Combining anti-PD-1 therapy with nano-drug composed of an Monocarboxylate transporter 1 (MCT1) inhibitor loaded inside the ultra-pH-sensitive nanoparticles increases the effect of T cells and decreases exhaustion of T cells, significantly reducing tumor volume and prolonging survival (72). And the effect of nanoparticle treatment is attributable to alleviating the acidic microenvironment further activating T cells (86). The therapeutic implication of these models is that the application of nanomaterials in targeting metabolism can accurately target tumor lesions to enhance the immune response.

### Combination of Targeting Metabolic Disorders and Immunotherapy in Clinical Therapeutics

With research continuing to reveal the effect of metabolic alteration on tumor immune response, clinical trials are worth exploring. The improved outcomes have been observed after combined ICB and metformin treatment compared with single ICB in melanoma (102), small cell lung cancer (103), and non-small cell lung cancer (104). As preclinical findings that inhibition of indoleamine 2,3-dioxygenase (IDO), a principal enzyme in tryptophan catabolism, could enhance the immune response in the mouse model show promise (105), the study investigates whether it still works in clinical trials. However, combing IDO inhibitor with anti-PD-1 therapy failed to prolong patients’ survival compared with immunotherapy alone in melanoma (106). Although interference with specific metabolic pathways in tumor cells or immune cells can be synergistic with immunotherapy, these targets may not be applicable in the current tumor lesion. Thus, studies need to continue to explore metabolic flexibility and metabolic adaptability based on the different cell functions in various types of cells within the current hypoxia TME.

## Exploring Metabolic Plasticity Under Hypoxia Tumor Microenvironment in the Single-Cell Era

As the cellular metabolic heterogeneity and hypoxia level *in vitro* cultures are far from the physiological characteristics of tumor lesions, the complexity of TME in cancer patients’ tissues is hard to be fully revealed in the past. The biggest technological advances in recent years of single-cell approaches can be applied to analyze and provide the more faithful biological and metabolic characteristics of cells at single-cell resolution (**Figure 1**).

### Revealing Cellular Metabolic Plasticity and Immunity Function Based on a Single-Cell Study

Recently, various methods developed for studying single cells have been used to reveal the metabolism of cells derived from tissues in cancer patients (107–109). A study based on single-cell metabolic regulome proteins defines T-cell subtypes using 27 metabolisms and 18 cellular phenotypic protein expressions and reveals that CD8^+^ T cells with low metabolism are at the edge of the tumor, while CD8^+^ T cells with high metabolism are near the core of the tumor (110). Miller et al. (111) developed a method based on enzyme activity on consecutive slides to evaluate the metabolism of cells in cancer tissues and find glycolytic Glyceraldehyde-3-phosphate dehydrogenase (GAPDH) activity enhanced in CD8^+^ T cells but reduced in Tregs in human colon cancer compared with normal tissues. This study is inconsistent with the earlier study (43) that Treg is inclined to use glycolysis and fatty acid to provide energy for cell activity, suggesting elusive shapes of TME on the metabolism of immune cells. A study uses SCENITH, a method for metabolic profiling samples, with scRNA-seq to explore the metabolism characteristics of myoid cells in cancer. Interestingly, they find TAMs behave highly TCA metabolism in renal carcinoma tumor tissues but result in high glycolysis in tumor-adjacent tissues, while monocytes and DC cells prefer glycolysis in both tumor and adjacent tissues (112), which is consistent that M2-like TAMs prefer to use TCA to maintain cell viability. This study shows that the metabolic function of TAMs is specifically regulated by TME through affecting metabolic gene expression and also implies that the metabolism gene expression reflates metabolism activity to a great extent. The RNA-seq data of metabolic gene expression indeed reflect the metabolic situation, which has been confirmed by bulk and scRNA-seq analysis (112, 113). The scRNA-seq data reveal the positive correlation not only between hypoxia and glycolysis but also between hypoxia and OXPHOS in the tumor, stromal and immune cells across squamous cell carcinoma of the head and neck and melanoma (114). The high fatty acid level in TME leads to increased uptake of fatty acid metabolism in tumor cells and results in lipid scarcity to damage T-cell function, and reducing fatty acid uptake by manipulating key gene expression in tumor cells would enhance the antitumor immunity in the mouse model study (115).

### Revealing Antitumor Immunity Under Hypoxia Tumor Microenvironment Using Single-Cell Study

Hif1a-deficient NK cell subcluster enhances the OXPHOS and antitumor ability in lung cancer mouse model observed by scRNA-seq study, indicating that regulation of hypoxia-induced pathways is a way to resist the damage caused by hypoxia (39). Based on the single-cell analysis in human liver cancer, tumors with higher hypoxia levels show higher Treg infiltration and reduced expression of cytotoxicity-related genes in CD8^+^ T cells than in low-diversity hypoxia (116), implying that hypoxia might influence the immune ability for antitumor through to bring multiple cell types. Interestingly, elevated HIF1A in TAMs is significantly associated with resistance to antiangiogenic therapy based on scRNA-seq analysis on Clear cell renal cell carcinomas (ccRCCs) patients (117), indicating that hypoxia TME is essential for the protumor function of TAMs. Single-cell and bulk RNA-seq analysis shows that HIF1A-inhibited tumor cells can increase glycogen synthesis and lead to an inflammatory response that contributes to tumor formation in pancreatic tumors (118). Our earlier study based on scRNA-seq shows that hypoxia level is positively correlated with gene signature and gene expression related to T-cell exhaustion. Also, SPP1+TAMs, potentially enhancing tumor metastasis and immunosuppression, are observed to be highly expanded in hypoxia TME across independent patient samples in six cancer types (119).

### Rethinking After Learning From Single-Cell Studies

As shown above, with the development of single-cell technologies, we can reveal the previous missed immune cell subtypes and their corresponding metabolic state in patient samples, which provides a clearer view of metabolic changes within hypoxia TME. For example, SPP1+TAMs, which are different from the classic classification of TAMs and accounts for a large proportion of macrophages within the tumor, have a tumor-promoting phenotype under hypoxic conditions (119). Besides, the metabolic characteristic of different cell subtypes inspires three concepts that subvert the previous cognition. First, we cannot simply define hot or cold tumors based on the level of T-cell infiltration because the infiltrated T cells will be affected by the local hypoxia TME as well as the nutritional accessibility and have different metabolic activities and immunophenotypes (110). Second, the metabolic profile of immune cells in normal tissues may not reflect their metabolic activity in cancer because different myeloid cell subtypes have distinct metabolic patterns in the same TME but the same in adjacent tissues (112). Third, previous conclusions are too simplistic for cancer to analyze the complex TME, as there is a high correlation between hypoxia and glycolysis as well as OXPHOS in tumors (114). These results further indicate that integrating single-cell approaches is important in advancing metabolic studies of immune cells, characterizing metabolic flexibility within a cell subtype and well quantifying metabolic heterogeneity among different cell types in the current TME. Indeed, with the improvements of scRNA-seq technology and accumulated datasets, the computational biology method for studying metabolism based on scRNA-seq needs to be developed to characterize cancer metabolism to characterize the metabolic diversity in a single-cell landscape (120). Besides, the metabolic findings should also be validated by using multiple strategies and a large sample.

## Discussion, Conclusion, and Outlook

Overall, we now recognize that the success of antitumor therapies is widely influenced by insufficient oxygen, metabolism reprogramming, and by-products induced by hypoxia in the local TME; therefore, interpreting how these processes specifically influence immune cell function could be applied in immunotherapy. Based on such observations, we put forward a concept of “understanding and regulating cellular plasticity to the current TME,” stating that the phenotype of immune cells to the current TME situation includes hypoxia and nutritional deficiency. Exploring which characteristics of the TME have the greatest impact on immune cells, then blocking pathways leading to the current TME, will alleviate harsh TME and selectively affect the protumor cells demand those pathways and enhance the antitumor function of effector cells. Revealing the flexible metabolic pathways activated in specific cell types within hypoxia TME based on clinical samples is of great significance for precise targeting. As multi-omic datasets accumulated, reasonable and efficient use of these data will provide a good value for studying metabolic plasticity of immune cells and corresponding cell state within the hypoxia TME. Cytometry and scRNA-seq are complementary approaches, and the metabolic flexibility of each cell type can be analyzed and inferred from the results of scRNA-seq through key gene expression with specific metabolic pathways and networks and then verified through cytometry. For example, one can collect the key metabolism feature of T-cell exhaustion subtypes in hypoxia TME by analyzing published scRNA-seq data and then use a few specific key metabolic antibodies used for flow cytometry and further provide more precise metabolic reprogramming under hypoxia or nutritional accessibility. Of course, there are few computer methods and tools available to present the metabolic landscape at the single-cell level; therefore, additional development and improvement of methods and analytical tools, including clustering methods using metabolic features on the single-cell level and pathway enrichment techniques for single cells, are indispensable.

## Author Contributions

JW wrote the original draft of the article. MH made contributions to making figures and organizing the table. HD reviewed and revised the article. All authors approved the final version of the article.

## Funding

This work was funded by the National Key R&D Program of China (2018YFC0910201) and the Key R&D Program of Guangdong Province (2019B020226001).

## Conflict of Interest

The authors declare that the research was conducted in the absence of any commercial or financial relationships that could be construed as a potential conflict of interest.

## Publisher’s Note

All claims expressed in this article are solely those of the authors and do not necessarily represent those of their affiliated organizations, or those of the publisher, the editors and the reviewers. Any product that may be evaluated in this article, or claim that may be made by its manufacturer, is not guaranteed or endorsed by the publisher.
